# Canine Distemper Outbreak in Rhesus Monkeys, China

**DOI:** 10.3201/eid1708.101153

**Published:** 2011-08

**Authors:** Wei Qiu, Ying Zheng, Shoufeng Zhang, Quanshui Fan, Hua Liu, Fuqiang Zhang, Wei Wang, Guoyang Liao, Rongliang Hu

**Affiliations:** Author affiliations: Center for Disease Control and Prevention, Chengdu Military Region, Kunming, People’s Republic of China (W. Qiu, Y. Zheng, Q. Fan, H. Liu, F. Zhang, W. Wang, G. Liao);; The Veterinary Research Institute of Academy of Military Medical Sciences, Changchun, People’s Republic of China (S. Zhang, R. Hu)

**Keywords:** canine distemper, canine distemper virus, rhesus monkeys, epidemic, measles-like infection, viruses, People’s Republic of China, dispatch

## Abstract

Since 2006, canine distemper outbreaks have occurred in rhesus monkeys at a breeding farm in Guangxi, People’s Republic of China. Approximately 10,000 animals were infected (25%–60% disease incidence); 5%–30% of infected animals died. The epidemic was controlled by vaccination. Amino acid sequence analysis of the virus indicated a unique strain.

Canine distemper is a highly contagious infectious disease of canine and feline species caused by canine distemper virus (CDV), a member of family *Paramyxoviridae* (*1*). Susceptible animals include dogs, wolves, jackals, foxes, mongooses, badgers, raccoon dogs, skunks, minks, and ferrets (*2**–**6*). Case-fatality rates for these animals has ranged from 30% to 80% and even to 100% of ferrets (*7*). Natural infection with CDV has occasionally been reported in bears, lesser pandas, and giant pandas (*8**–**10*). Monkeys are not generally considered susceptible but can be experimentally infected (*11**,**12*). In 1989, the first natural case of canine distemper in a monkey (*Macaca fuscata)* was reported (*13*). Recently, natural canine distemper infection was reported in a few monkeys in Beijing, People’s Republic of China, with a description of the clinical signs and pathogenic changes (*14*). This outbreak most likely resulted from secondary transmission of CDV originating in a larger outbreak on a Guangxi breeding farm, where a similar disease had occurred 2–3 years earlier. Here we describe this larger outbreak and provide a more detailed epidemiologic analysis.

## The Study

In 2006, an unidentified respiratory disease occurred in rhesus monkeys (*Macaca mulatta*) at a breeding farm in the Guangxi Zhuang Autonomous Region in southern China. The farm, the largest in China, comprised 31,260 monkeys, of which ≈1 in 5 was unweaned. Approximately 10,000 monkeys contracted the disease, and 4,250 died. The morbidity rate in young monkeys was 60%, with an ≈30% death rate (25% and 5%, respectively, for adults). In 2007, surviving monkeys were vaccinated with an inactivated suspension made from the livers and lungs of dead animals. After vaccination, the number of cases decreased during 2007 and 2008 to ≈100–200 per year.

Cases occurred throughout 2006. Because most authorized suppliers of monkeys to research laboratories in China obtain their breeding stock from this farm, the disease spread throughout China, particularly to experimental animal facilities in Wuhan, Kunming, and Beijing (*14*). The disease also was introduced into a few wildlife parks in China; however, perhaps because of the low population density of susceptible animals in these locations, further spread has not been reported.

Initially, CDV was not suspected as the causative agent of the monkeys’ illness. In late 2008, however, tissue specimens from infected animals that had been stored in a freezer for 2 years were analyzed and found to contain CDV. Four serum samples from adult monkeys whose illnesses had naturally resolved had titers of 4–32 virus neutralizing antibodies against CDV, whereas virus neutralizing antibodies could not be detected in 3 serum samples from uninfected monkeys. After this identification, all experimental monkeys were vaccinated with attenuated CDV vaccine starting in early 2009. Whether the vaccines actually boosted immunity or whether they were simply given coincident with waning of the outbreak from increasing immunity, the number of cases has since remained low (≈130 in 2009 and 20–30 in 2010 [not all confirmed]). Additionally, anti-CDV serum seemed to help infected animals recover more rapidly from the infection.

**Figure 1:**
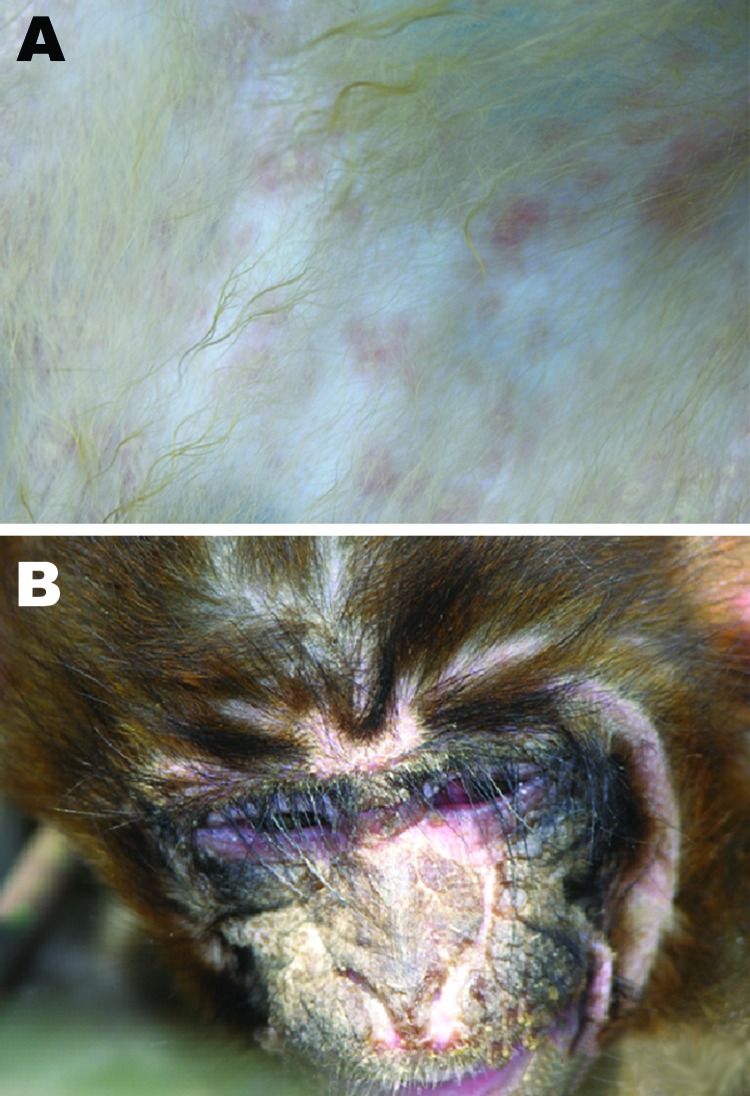
Canine distemper virus signs in rhesus monkeys at necropsy. A) Rash; B) suppurative conjunctivitis.

Infected monkeys initially displayed measles-like signs, including respiratory signs; anorexia; fever; and red rashes over the entire body, with reddening and swelling of the footpads; conjunctivitis; and thick mucoid nasal discharge. Coma preceded death. Postmortem examination demonstrated discrete purple or rosy rashes on the body, with macules of 2–4 mm (Figure 1, panel A), which in some cases were confluent and had a dark rosy color. Rashes on the face were vesicular and ulcerated after suppuration, forming a scab. Other signs included congestion, redness and swelling of the pars oralis pharyngis, diffuse hemorrhagic spots on the papillae of the tongue, suppurative conjunctivitis (Figure 1, panel B), and rhinitis with copious thick mucous exudates. Blood stasis patches in the lungs and interstitial fibrosis were observed in most affected lungs. Infected monkeys also had blood stasis in parts of the liver, with tiny khaki-colored hemorrhagic spots on the surface.

Because of the signs of a measles-like infection, identification of the etiologic agent first focused on measles virus and later on CDV. Reverse transcription PCR amplification of lung specimens by using virus-specific primers was negative for measles virus but positive for CDV. The supernatant of ground liver samples also was positive for CDV by immunochromatographic analysis (BIT Rapid Color CDV cassette, Bioindist Co. Ltd., Yong-in City, Gyeonggi-do, South Korea). Lung specimens also infected tree shrews (1–2 months old) after subcutaneous injection, resulting in excitement and death from encephalitis and enterohemorrhage.

**Figure 2:**
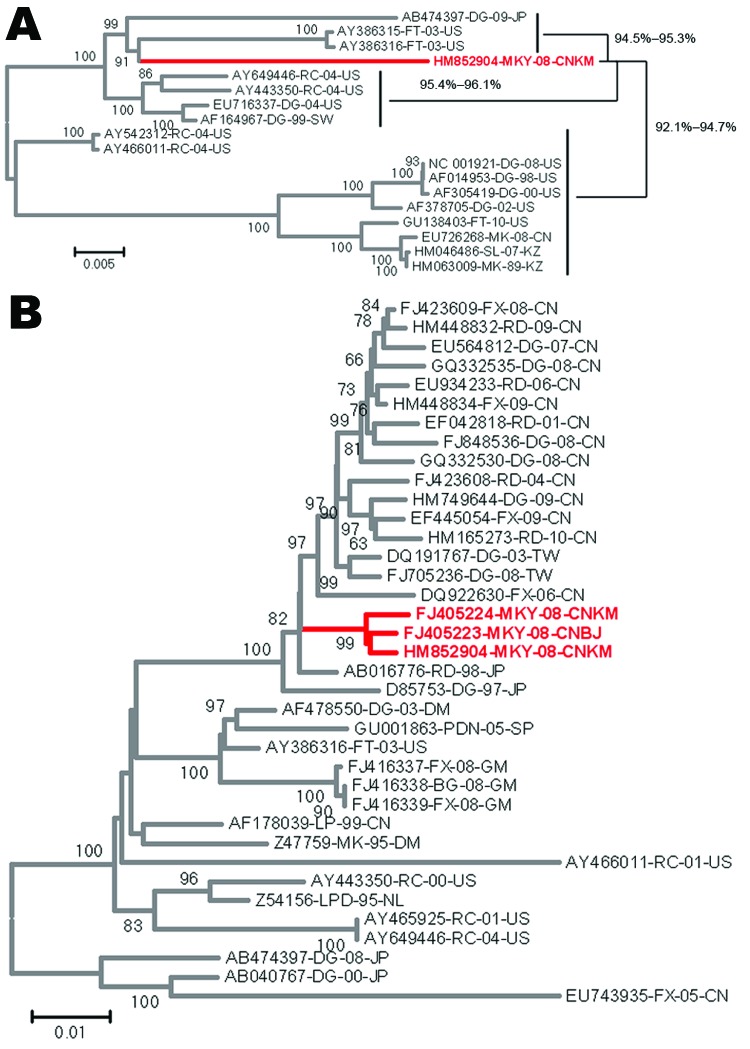
Phylogenetic analysis of the canine distemper virus by comparison of the genome or gene of the monkey isolate with other canine distemper virus isolates. A) Full genome. B) H gene. FX, fox; CN, People’s Republic of China; RD, raccoon dog; DG, dog; TW, Taiwan; MKY, monkey; CNKM, Kunming, People’s Republic of China; CNBJ, Beijing, People’s Republic of China; JP, Japan; DM, Denmark; PDN, *Lynx pardinus*; SP, Spain; FT, ferret; US, United States; GM, Germany; BG, badger; LP, lesser panda; MK, mink; RC, raccoon; LPD, leopard; NL, the Netherlands. Scale bars indicate phylogenetic distance between isolates.

The full-length viral genome was amplified and sequenced (GenBank accession no. HM852904). We constructed phylogenetic trees by the neighbor-joining method, using the full sequence of this virus and others available in GenBank. The full genome of this isolate shared the highest homology with a ferret isolate (accession no. AY386316) from the United States, a raccoon isolate (accession no. AY649446) from the United States, and a dog isolate (accession no. AB474397) from Japan (Figure 2, panel A). The L gene showed the highest identity (95.6%–96.5%) with that of US ferret and raccoon isolates (accession nos. AY386316 and AY466011); phylogenetic analysis of the H gene indicated an eastern Asian source of this isolate, which shared high homology with isolates from different species of animals in China, Taiwan, and Japan (Figure 2, panel B). Overall, the amino acid homology of the Guangxi isolate with the others was 96.0%–97.3%, clustering it in a large clade that includes CDV isolates from Asia. Nevertheless, the isolate is unique in that it contains multiple amino acid changes in its viral structural proteins, none of which have been found in other isolates.

Reasons for the epidemic remain unclear. The first monkey to contract the infection was in the farm in Guangxi; however, the source of the infection is unknown because there were no dogs or other fur-bearing animals at the farm. Breeding facilities were self-contained, with no introduction of external animals and no other farms nearby. Food for the monkeys had no animal content except fish powder. One possible source of infection is contact by the monkeys at the farm with local wild monkeys. Another possibility is spillover of the virus from a stray dog carrying CDV that became adapted to the new host.

Large-scale breeding and caging of the monkeys might have contributed to increasing susceptibility to canine distemper infection. Reared in groups of 20–30 within fenced-off areas or in pens of several hundred, close contact between animals was inevitable. CDV appears to have been transmitted by droplets derived from feces or other body discharges, all of which contained CDV.

This canine distemper outbreak poses a threat to monkey populations. Because the Guangxi farm routinely supplies monkeys for animal facilities throughout China, monitoring for canine distemper in its monkeys, including those shipped to other animal facilities, as well as human handlers, is advisable to prevent possible secondary spread and interspecies transmission.

## Conclusions

Although CDV spread has been largely controlled by inactivated and live canine distemper vaccines, sporadic cases still occur and the high number of mutations in the virus makes future transmission unpredictable. Therefore, surveillance for canine distemper should be considered among monkey populations and among humans who have close contact with them.
